# Epidemiology of pharmaceutically treated depression and treatment resistant depression in South Korea

**DOI:** 10.1371/journal.pone.0221552

**Published:** 2019-08-23

**Authors:** Namwoo Kim, Sung Joon Cho, Hyeyoung Kim, Se Hyun Kim, Hyun Jeong Lee, C. Hyung Keun Park, Sang Jin Rhee, Daewook Kim, Bo Ram Yang, So-Hyun Choi, GumJee Choi, MinJung Koh, Yong Min Ahn

**Affiliations:** 1 Department of Neuropsychiatry, Seoul National University Hospital, Seoul, Republic of Korea; 2 Department of Psychiatry, Kangbuk Samsung Hospital, Sungkyunkwan University School of Medicine, Seoul, Republic of Korea; 3 Department of Psychiatry, Inha University Hospital, Incheon, Republic of Korea; 4 Department of Psychiatry and Behavioral Science, Seoul National University College of Medicine, Seoul, Republic of Korea; 5 Mental Health Clinic, National Cancer Center, Goyang-si, Republic of Korea; 6 Medical Research Collaborating Center, Seoul National University Hospital, Seoul, Republic of Korea; 7 Medical Affairs, Janssen Korea, Seoul, Republic of Korea; Iwate Medical University, JAPAN

## Abstract

**Background:**

The epidemiology of pharmaceutically treated depression (PTD) and treatment resistant depression (TRD) is largely unknown in South Korea. The aim of this study was to develop a greater understanding of the characteristics of PTD and TRD in nearly the entire adult population in South Korea using the Health Insurance Review and Assessment Service (HIRA).

**Method:**

Diagnostic codes and prescription data for South Korean adults were extracted from the HIRA. Subjects were included in the PTD cohort if they received at least one prescription for antidepressants and were diagnosed with depression. TRD was defined as PTD having two or more regimen failures of antidepressants or antipsychotics.

**Results:**

In 2012, there were 41,256,396 adults in South Korea with 834,694 meeting the criteria for PTD (2.0%). Among subjects with PTD, 57% stopped treatment in less than 28 days of antidepressant supply. Tricyclic and tetracyclic antidepressants were the most frequently used antidepressants as a first-line regimen for PTD (44.3% of PTD) followed by selective serotonin reuptake inhibitors (32.1% of PTD). Results also indicated that 34,812 subjects developed TRD (4.2% of PTD). Median PTD and TRD durations were 28 and 623 days respectively. Proportions of psychiatric and non-psychiatric comorbidities were higher in TRD cases than in PTD cases that were not treatment resistant.

**Conclusions:**

Despite a small proportion of patients with TRD, the prolonged duration of illness and higher comorbidity implies the need for better treatment.

## Introduction

Major depressive disorder is a common worldwide condition, with 12-month prevalence rate of 6.7% in the United States [1]. Despite effective pharmacological treatments, approximately only 30% of patients were in remission following 12 weeks of antidepressant treatment, as reported by the study of Sequenced Treatment Alternatives to Relieve Depression (STAR*D) [2]. Furthermore, approximately 33% of patients with depression failed to achieve remission even after four consecutive trials of antidepressant treatment [3].

Treatment resistant depression (TRD) is common [2, 4] and has been associated with higher comorbidities [5] and prolonged duration of illness [6], leading to a substantial medical and economic burden [7–9]. Despite the ongoing disputes over a precise TRD definition, a general consensus identifies TRD when at least two adequate trials of antidepressants with different mechanisms of action fail to achieve remission [10, 11]. The adequate trial represents using effective dosage with sufficient duration of antidepressant treatment, however, there is a variety of standards regarding daily dosage and duration [8, 12]. The need for antidepressants with different mechanisms of action in TRD is also under debate due to inconsistent results on the effectiveness of switching to antidepressants in the same class or a different one [10, 13–15].

Moreover, in clinical settings, there are substantial off-label prescriptions of atypical antipsychotics as an adjunctive treatment for TRD [16]. Results of a meta-analysis indicated that augmenting antidepressants with atypical antipsychotics for TRD is more effective than antidepressants with placebo [17]. Therefore, not only antidepressants, but also atypical antipsychotics need to be considered when evaluating failed adequate trials of treatment in TRD. According to the diverse arguments discussed above, the varied reported rates of TRD are: 2.47% [18], 6.6% [8], 12% [19], 29% [20], and 35% [12].

Little is known about the epidemiology of TRD in countries of the Asia-Pacific region including South Korea. To understand the epidemiology of TRD in South Korea, the current study used retrospective healthcare data from nearly the entire population of South Korea. The primary objective of this study was to describe the epidemiological outcomes of pharmaceutically treated depression (PTD) and TRD of South Korean adults, including the prevalence, incidence and illness duration by age and sex. The secondary objectives included determining the proportions of psychiatric and non-psychiatric comorbidities, and the frequency of prescribed antidepressants and antipsychotics in patients with PTD and TRD.

## Materials and methods

### Data source

We collected diagnostic codes and prescriptions of medications for each person using the Health Insurance Review and Assessment Service (HIRA), which contains claims data from a universal healthcare system that covers approximately 98% of the South Korean population—National Health Insurance [21, 22]. Data collected between January 1, 2011 and December 31, 2015 were retrospectively reviewed.

### PTD cohort

This study focused on subjects with depression who did not respond to pharmaceutical treatment. Therefore, subjects with depression who did not undergo antidepressant treatment or underwent treatments other than antidepressants, such as cognitive behavioral therapy, were not included in this study.

**Fig 1:**
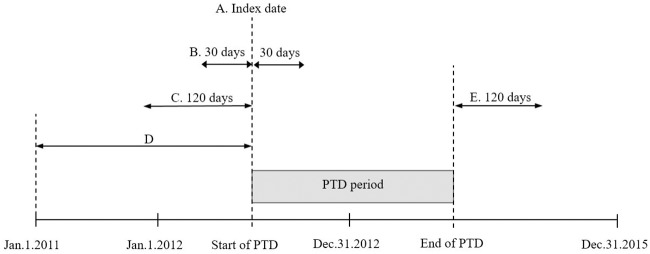
Subject selection and pharmaceutically treated depression (PTD) period. Several conditions were required for subjects to be included in this study. A: The Index date is the first date of a dispensing of an antidepressant within 2012. Subjects were aged ≥ 18 years on the index date. B: Subjects were diagnosed with depression within 30 days either before or after the index date. C: Subjects had not received an antidepressant medication during the last 120 days before the index date. D: Subjects had not received an exclusion diagnosis during this period. E: PTD ended when: subjects had 120 days of no dispensing of an antidepressant, met the exclusion criteria, or reached the end of the study period (December 31, 2015).

A subject’s depressive episode was estimated based on the period for which the subject was dispensed antidepressants as limited information regarding patients’ clinical symptoms were reported; the estimated depressive episode was defined as PTD. Subjects were included in the PTD cohort if they (1) received at least one antidepressant prescription in 2012, (2) were aged ≥ 18 years on the index date; the index date of PTD was defined as the first date of receiving an antidepressant prescription in 2012, (3) had not received an exclusion diagnosis between January 1, 2011 and the index date, and (4) had not received an antidepressant during the last 120 days before the index date, i.e., we focused on newly developed depressive episodes that began in 2012, rather than ongoing episodes. PTD began on the index date and ended when: the subject had not been dispensed an antidepressant for 120 days, met the exclusion criteria, or reached the end of the study period (December 31, 2015) (Fig 1). Depressive episodes with onset after January 1, 2013 were not included as PTD onset needed to occur in 2012. Only the first PTD occurrence was analyzed in each subject and follow up was conducted until December 31, 2015.

Diagnoses of depression according to the ICD-10 code included: depressive episode (F32), recurrent depressive disorder (F33), and dysthymia (F34.1); these diagnoses did not need to be a primary diagnosis. While subjects classified by these diagnostic codes are heterogeneous, they share a common feature in that the severity of their depressive symptoms were likely high enough for them to be prescribed antidepressants. Subjects were excluded if they received any of the following diagnoses: schizophrenia (F20), schizotypal disorder (F21), persistent delusional disorders (F22), acute and transient psychotic disorders (F23), schizoaffective disorders (F25), other nonorganic psychotic disorders (F28), unspecified nonorganic psychosis (F29), manic episode (F30), bipolar affective disorder (F31), dementia in Alzheimer disease (F00), vascular dementia (F01), dementia in other diseases classified elsewhere (F02), unspecified dementia (F03), or organic amnesic syndrome, not induced by alcohol and other psychoactive substances (F04). Manic episode (F30) and bipolar affective disorder (F31) were only considered as exclusion diagnoses if they were given as a primary diagnosis; in South Korea, if a patient with depressive disorder needs to have a mood stabilizer, psychiatrists often add a diagnosis of bipolar disorder as a non-primary diagnosis due to a medical insurance issue, even if there is no definitive evidence of bipolar disorder. Although this procedure confused the design of the current study, these criteria allowed for a more accurate representation of the clinical situation in South Korea.

### Regimen of antidepressants and antipsychotics

As described above, the definition of TRD was often based on failures of at least two adequate trials of antidepressants. In this study, we defined an adequate trial of an antidepressant as an antidepressant regimen which occurred during PTD for at least 28 days’ supply (minimum of 4 weeks) with no breaks of more than 30 days between the expected end day of the supply to the start date of the next dispensing of the same medication, i.e., the antidepressant regimen required only the ‘sufficient duration of treatment’. We did not set a minimum dose requirement for an antidepressant regimen because in South Korea, it is common for psychiatrists to prescribe antidepressants in doses less than the minimum effective daily dose [23] due to side effects. Therefore, we hypothesized that a regimen without a minimum dose requirement better reflected real clinical practice. Also, in South Korea, psychiatrists determine the effectiveness of previously prescribed antidepressants, usually within 2 to 6 weeks, before switching to other medications. Since there is no absolute standard for the minimum duration of maintaining medication, we repeated the analysis with three different sets based on 2, 4, or 6 weeks as a minimum duration of a medication regimen.

Previous meta-analyses have shown that augmenting antidepressants with atypical antipsychotics in patients with TRD improves depressive symptoms [7, 24]. Therefore, it is reasonable to include antipsychotics regimens. Regarding antipsychotics regimens, we selected four frequently used antipsychotics for treatment of depression in South Korea: olanzapine, aripiprazole, amisulpride, and quetiapine. In contrast to the antidepressant regimen, since antipsychotics with low dose were frequently used to alleviate sleep problems, we set a minimum dosage requirement for an antipsychotics regimen as follows: olanzapine 5mg/day [25], aripiprazole 2mg/day [26, 27], quetiapine 50mg/day, and amisulpride 50mg/day. Each antipsychotic medication was considered as a regimen only if it was augmented with antidepressants, i.e., if a patient had antipsychotics without an antidepressant in the same day, that day was not counted toward the required minimum days of medication supply for a regimen of antipsychotics. A regimen of antipsychotics, by definition, could not be the first regimen of PTD (i.e., the first regimen of PTD was always antidepressants).

A regimen ended when there was no dispensing of the medication for 30 days. A regimen failed if a new regimen was begun, i.e., starting a new regimen implied that the earlier regimen was inadequate. Furthermore, if a regimen was maintained for more than 90 days, we assumed that the regimen was effective and we defined that the regimen cannot be a failed one. This threshold was set based on clinical practice.

An earlier regimen was considered to fail on the date when a later regimen began; although the earlier regimen could still have continued. The new regimen was not required to be in a different class as there is lack of sufficient evidence indicating that switching medication to a different class leads to better outcomes [28, 29]. If two regimens started simultaneously, we assumed that it was due to one regimen not being sufficiently able to improve depressive symptoms; therefore, in such a situation, the sum of the failed regimens was incremented by one.

### TRD

The definition of TRD is PTD with at least two failed regimens. The incident date of TRD was the date when the second regimen failed. The definitions of the regimen, PTD, and TRD are similar to those in a recent study conducted in Taiwan [18].

### Psychiatric and non-psychiatric comorbidities

We considered the following disorders as psychiatric comorbidities: anxiety disorders, substance related disorders, obsessive-compulsive disorder, and personality disorders. Non-psychiatric comorbidities included: cardiovascular diseases, diabetes mellitus, chronic obstructive pulmonary diseases, cancer, stroke, and hypothyroidism. The ICD-10 code for each comorbidity is listed in S1 Table. If a comorbidity was diagnosed at least once during PTD, the PTD was considered to have a comorbid disorder.

### Statistical analysis

The prevalence of PTD was calculated as the number of PTD patients divided by the population of South Korean obtained from 2012 Population Statistics data based on resident registration from Statistics Korea [30]. We calculated the proportion of TRD to PTD (number of TRD patients /number of PTD patients), and the incidence of TRD per 100 person-years, by dividing the number of TRD patients by the total number of person-years and multiplying the result by 100. The 95% confidence intervals were calculated assuming a Poisson distribution. Chi-square tests were performed for comparison of psychiatric and non-psychiatric comorbidities between TRD and PTD without TRD. All data were analyzed using SAS Enterprise Guide, version 6.1 (SAS Institute, Cary, NC, USA), and *p*-values less than 0.05 were considered statistically significant.

### Ethical considerations

All personal information in the HIRA was preprocessed into unidentifiable codes, thus, informed consent was not required. This study protocol was exempted from review by the Institutional Review Board of Seoul National University Hospital and the Seoul National University College of Medicine (IRB number: 1611-056-807).

## Results

### PTD cohort

**Fig 2:**
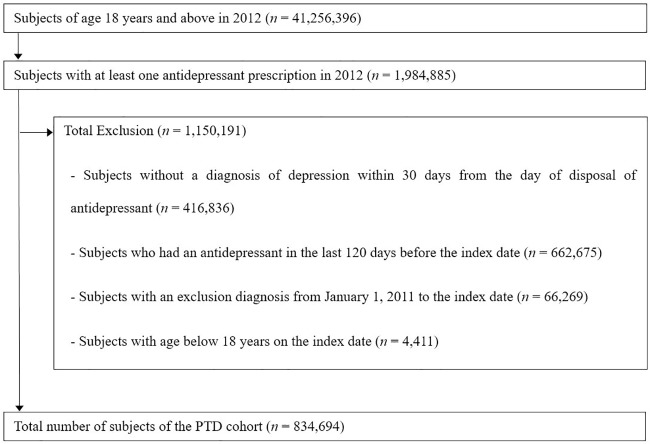
Pharmaceutically treated depression (PTD) selection flow.

As shown in Fig 2, 41,256,396 people were aged 18 years and above in South Korea in 2012 and, 1,984,885 subjects (4.8% of 41,256,396) had been prescribed antidepressants at least once during the year. From those prescribed subjects, 834,694 (2.0% of 41,256,396) were determined as the total PTD cases for this study. In other words, 2% of the adults in South Korea who were newly diagnosed with depressive disorder received antidepressant treatment in 2012. Among the patients who received at least one antidepressant prescription, 21.0% of them had no diagnosis of depression within 30 days from the date of the antidepressant prescription. The most common diagnosis of those patients was ‘other anxiety disorder’ (F41), which was observed in 11.1% of them (S2 Table).

### Number and duration of regimens

From the 834,694 PTD cases, 57.0% did not have a regimen at all (Table 1) and 98.8% of PTD cases had no regimen of antipsychotic medications. Therefore, approximately 43.0% and 1.2% of PTD cases had at least one regimen of antidepressants and antipsychotic medication, respectively. The mean number of regimens of antidepressants and antipsychotics was 0.8 and 0.0, respectively. The median number of regimens of antidepressants and antipsychotics were both 0. The mean and median duration of regimens of antidepressants were 174.1 and 78 days, respectively. The duration of antipsychotic regimens was similar to that of antidepressants, due to the definition of the antipsychotics regimen described in the method section.

**Table 1:** Number and duration of regimens of PTD.

Number of regimens	PTD cases (*n* = 834,694)		
	Antidepressants (%)	Antipsychotics (%)	All (%)^a^
0	476,086 (57.0)	824,767 (98.8)	476,086 (57.0)
1	214,783 (25.7)	7,655 (0.9)	211,980 (25.4)
2	73,224 (8.8)	1,530 (0.2)	73,748 (8.8)
3	27,294 (3.3)	442 (0.1)	27,853 (3.3)
≥ 4	43,307 (5.2)	300 (0.0)	45,027 (5.4)
Mean ± std	0.8 ± 1.6	0.0 ± 0.2	0.9 ± 1.6
Median (min,max)	0 (0,48)	0 (0,14)	0 (0,48)
Duration of regimens: days			
Mean ± std	174.1 ± 247.8	172.5 ± 216.9	174.1 ± 247.2
Median (min,max)	78 (28,1461)	90 (28,1456)	78 (28,1461)

^a^ Number of subjects who had antidepressants or antipsychotics regimens.

### Prevalence of PTD and TRD by age and sex

Table 2 shows that the prevalence of PTD in males and females was 1.42% and 2.62%, respectively, thus, the prevalence of PTD was 1.85 times higher in females than males. PTD prevalence increased with age with the exception of people over 80 years old in both sexes. There was a total of 34,812 TRD cases and the prevalence of TRD in the general population was 0.08%. The proportion of TRD to PTD was 4.17%, and it was highest in males aged 18 to 29 and in females in their 30s. The sex ratio of TRD was approximately equal to that of PTD, i.e., the proportion of TRD to PTD in males was approximately equal to that in females (1:1.00). The incidence of TRD was 11.5 events per 100 person-years and was inversely correlated with age in both sexes. We repeated the analyses by changing the requirement of minimum days of medication supply for a regimen to 14 and 42 days. The results indicated that the proportion of TRD to PTD was 6.61% (14-day-set) and 2.68% (42-day-set) (S3 and S4 Tables).

**Table 2:** Prevalence of PTD and prevalence, proportion, incidence of TRD according to age and sex.

Group	General population	PTD cases	PTD Prevalence (%)	TRD cases	TRD Prevalence (%)	Proportion of TRD to PTD (%)	sum of person year (PY)	TRD Incidence (95% CI)
All subject	41,256,396	834,694	2.02	34,812	0.08	4.17	302,657	11.5 (11.4,11.6)
Male	20,464,613	290,206	1.42	12,084	0.06	4.16	109,489	11.0 (10.8,11.2)
18–29	4,219,688	28,605	0.68	1,466	0.04	5.12	7,628	19.2 (18.3,20.2)
30–39	4,163,684	36,320	0.87	1,471	0.04	4.05	9,915	14.8 (14.1,15.6)
40–49	4,510,316	51,579	1.14	2,376	0.05	4.61	17,411	13.6 (13.1,14.2)
50–59	3,915,566	66,878	1.71	2,951	0.08	4.41	26,107	11.3 (10.9,11.7)
60–69	2,083,585	54,487	2.62	2,191	0.11	4.02	25,179	8.7 (8.3,9.1)
70–79	1,246,286	41,641	3.34	1,378	0.11	3.31	19,103	7.2 (6.8,7.6)
≥ 80	325,488	10,696	3.29	251	0.08	2.35	4,146	6.1 (5.3,6.9)
Female	20,791,783	544,488	2.62	22,728	0.11	4.17	193,168	11.8 (11.6,11.9)
18–29	3,831,374	45,223	1.18	1,949	0.05	4.31	9,859	19.8 (18.9,20.7)
30–39	4,005,259	62,259	1.55	3,408	0.09	5.47	18,307	18.6 (18.0,19.3)
40–49	4,325,842	92,647	2.14	4,135	0.10	4.46	28,959	14.3 (13.9,14.7)
50–59	3,876,004	134,871	3.48	5,695	0.15	4.22	45,596	12.5 (12.2,12.8)
60–69	2,244,951	100,667	4.48	4,106	0.18	4.08	42,364	9.7 (9.4,10.0)
70–79	1,724,057	83,637	4.85	2,934	0.17	3.51	37,944	7.7 (7.5,8.0)
≥ 80	784,296	25,184	3.21	501	0.06	1.99	10,139	4.9 (4.5,5.4)
Male: Female	1: 1.016	1: 1.88	1: 1.85	1: 1.88	1: 1.85	1: 1.00	1: 1.76	1: 1.07

PTD Prevalence = number of PTD cases / General population; TRD Prevalence = number of TRD cases / General population; Proportion of TRD to PTD (%) = (number of TRD cases / number of PTD cases) * 100; TRD Incidence (no. of event/100PY) = (number of TRD cases / total number of person-years) * 100

### Duration of PTD and TRD

The mean duration of PTD, PTD without TRD, and TRD was 152, 86, and 713 days, respectively (Table 3). The median duration of PTD, PTD without TRD, and TRD was 28, 21, and 623 days, respectively. Furthermore, the mean and median number of days from the PTD index date to the TRD incidence date were 259 and 153, respectively.

**Table 3:** Duration of PTD, PTD without TRD, and TRD and time from the index date to the incident date of TRD (days).

	Duration of PTD			Time from the index date to the incident date of TRD
	Total	PTD without TRD	TRD	
N	834,694	734,050	34,812	34,812
Mean ± std	152 ± 304	86 ± 161	713 ± 458	259 ± 277
Median	28	21	623	153
Q1, Q3	7, 123	7, 86	228, 1180	70, 347

### Psychiatric and non-psychiatric comorbidities

The psychiatric and non-psychiatric disorders accompanied by PTD and TRD are shown in S5 Table. Anxiety disorders were the most frequently observed psychiatric comorbidity in all three groups (PTD, TRD, and PTD without TRD). Among non-psychiatric comorbidities, cardiovascular diseases were the most common in all three groups. TRD had significantly higher proportion of all comorbid disorders than PTD without TRD.

### Medications of regimens

Regimens of antidepressants and antipsychotics are listed according to the order of regimen in Table 4. In the table, a single regimen indicates that only one regimen started, without any other simultaneously started regimens. A double regimen indicates that supply of two regimens started on the same day. In PTD cases, medications of tricyclic antidepressants (TCA) & tetracyclic antidepressants (TeCA) had the highest proportion (44.3%) of the first-line, single regimens, followed by selective serotonin reuptake inhibitors (SSRI) (32.1%). Among the first-line regimens, SSRI & SSRI was the most frequently used combination (3.2%) followed by SSRI & TCA (2.0%). In the second-line regimens, rankings of antidepressant classes as a single regimen were the same as in the first-line regimens. The prescription frequency for every antipsychotic medication in the second-line regimen was less than 1%, with quetiapine being the most common (0.8%) and amisulpride the least prescribed medication (0.1%).

**Table 4:** Regimens of PTD and TRD.

	PTD cases (*n* = 834,694) (Col %)		TRD cases (*n* = 34,812) (Col %)			
	1st-line regimen	2nd-line regimen	1st-line regimen	2nd-line regimen	3rd-line regimen	4th-line regimen
	358,608 (43.0)	146,628 (17.6)	34,812 (100.0)	34,812 (100.0)	34,812 (100.0)	15,853 (45.5)
Single regimen ^a^						
Antidepressants						
SSRI	115,262 (32.1)	35,743 (24.4)	9,848 (28.3)	5,852 (16.8)	7,521 (21.6)	2,397 (15.1)
TCA & TeCA	158,821 (44.3)	44,332 (30.2)	6,636 (19.1)	5,518 (15.9)	6,561 (18.8)	2,242 (14.1)
SNRI	17,799 (5.0)	8,563 (5.8)	1,533 (4.4)	1,633 (4.7)	2,546 (7.3)	710 (4.5)
SARI	22,665 (6.3)	12,995 (8.9)	2,297 (6.6)	3,237 (9.3)	3,117 (9.0)	904 (5.7)
NaSSA	6,595 (1.8)	3,369 (2.3)	777 (2.2)	837 (2.4)	1,377 (4.0)	356 (2.2)
NDRI	4,518 (1.3)	2,494 (1.7)	341 (1.0)	569 (1.6)	849 (2.4)	187 (1.2)
MAOI	159 (0.0)	53 (0.0)	11 (0.0)	5 (0.0)	8 (0.0)	1 (0.0)
Antipsychotics						
Amisulpride	0 (0.0)	104 (0.1)	0 (0.0)	36 (0.1)	36 (0.1)	6 (0.0)
Aripiprazole	0 (0.0)	914 (0.6)	0 (0.0)	279 (0.8)	461 (1.3)	104 (0.7)
Olanzapine	0 (0.0)	133 (0.1)	0 (0.0)	52 (0.1)	75 (0.2)	21 (0.1)
Quetiapine	0 (0.0)	1,105 (0.8)	0 (0.0)	343 (1.0)	608 (1.7)	149 (0.9)
Double regimen ^b^						
SSRI & SSRI	11,416 (3.2)	12,467 (8.5)	4,211 (12.1)	5,002 (14.4)	2,849 (8.2)	2,433 (15.3)
SSRI & SNRI	345 (0.1)	427 (0.3)	152 (0.4)	209 (0.6)	198 (0.6)	186 (1.2)
SSRI & TCA	7,205 (2.0)	7,959 (5.4)	2,713 (7.8)	3,271 (9.4)	2,087 (6.0)	1,807 (11.4)
SSRI & AP	1,396 (0.4)	1,606 (1.1)	506 (1.5)	667 (1.9)	491 (1.4)	407 (2.6)
Others	10,812 (3.0)	12,578 (8.6)	4,172 (12.0)	5,516 (15.8)	4,050 (11.6)	3,333 (21.0)
≥ Triple regimen ^c^	1,615 (0.5)	1,786 (1.2)	1,615 (4.6)	1,786 (5.1)	1,978 (5.7)	610 (3.8)

SSRI = Selective serotonin reuptake inhibitors; TCA = Tricyclic antidepressants; TeCA = Tetracyclic antidepressants; SNRI = Selective norepinephrine reuptake inhibitors; SARI = Serotonin antagonist and reuptake inhibitors; NaSSA = Noradrenergic and specific serotonergic antidepressants; NDRI = Norepinephrine-dopamine reuptake inhibitors; MAOI = Monoamine oxidase inhibitors; AP = Antipsychotics.

^a^ Only one regimen began, i.e., no other regimens simultaneously began.

^b^ Two regimens simultaneously began.

^c^ Three or more regimens simultaneously began.

In TRD cases, SSRI as a single regimen was the most common first-line regimen (28.3%), followed by TCA & TeCA (19.1%); this ranking continued to fourth-line regimen. The proportion of SSRI & SSRI as a double medication in the first-line regimen for TRD was the highest (12.1%), followed by SSRI & TCA (7.8%); this ranking also continued to the fourth-line regimen. The proportion of antipsychotics as a single medication in the second-line regimen in TRD was similar to that of PTD.

## Discussion

This study described the epidemiology of PTD and TRD by utilizing the HIRA—the claims database of South Korea’s healthcare system that holds information on almost the entire population. While there are limitations when directly comparing the proportion of TRD to PTD between studies due to the lack of a universal definition for TRD, it is important to consider recent retrospective epidemiological studies using healthcare databases. Kubitz and colleagues utilized a claims database of commercial insurers in the United States and reported that 6.6% of 47,654 treated episodes of depression were TRD [8]. In Taiwan, Fife and colleagues reported that 2.47% of PTD cases were TRD with a 6-month cap on time from onset of PTD to TRD [18]. In Japan, Mahlich and colleagues reported that the incidence of TRD from PTD within a year was 12.0%, thus, the proportion of TRD to PTD should be higher than 12.0% [31]. The reason for the higher proportion of TRD in Japan’s group than in Taiwan’s group may arise from the loose definition of TRD in the study from Japan. In short, the proportion of TRD to PTD from the current study (4.17%) was similar to that reported in the American and Taiwanese studies, but lower than that from the study conducted in Japan.

It is important to note that 57% of PTD cases did not have a regimen at all; in other words, the median number of regimens for PTD cases was 0. This might imply that more than half of the patients who were prescribed at least one antidepressant recovered from depressive symptoms within 4 weeks, or they discontinued treatment without sufficient improvement due to unidentified reasons, such as intolerance to medications or high medical costs. Because the claims database only provides information of diagnoses and prescriptions, further research is needed to determine the reason why patients quit their medication before its maximum effect emerged.

A small proportion (1.2%) of PTD cases had at least one antipsychotics regimen. Therefore, in real-world practices in South Korea, using doses higher than the recommended minimum dose of antipsychotics to treat depressive disorder as augmentations with antidepressants appears uncommon. This may be partially caused by the required minimum dosage of antipsychotics for a regimen.

It has been shown that the onset of depression at an early age (younger than 18 years of age) is associated with TRD [32, 33]. In this study, although children and adolescents were not included, the incidence of TRD was inversely correlated with age. This contrasts PTD prevalence, which was positively correlated with age. There are several potential explanations for this: 1) younger patients with bipolar disorder may have been misdiagnosed with unipolar depressive disorder, thus multiple failures of various antidepressants resulted in TRD; 2) older patients may have a higher prevalence for non-psychiatric comorbidities and thus, might be prescribed antidepressants to prevent exacerbation of secondary mental illness. In such cases, the prophylactic use of antidepressants is usually maintained without switching to other medications and, therefore, the chance of failing is low; 3) older patients have a higher probability of having previous episodes of depression than younger patients. Previous episodes of depression can inform clinicians of the effectiveness of specific antidepressants, and therefore, help in determining the appropriate antidepressant and avoiding multiple trials of ineffective or intolerable antidepressants.

Patients with TRD had significantly higher proportion of psychiatric and non-psychiatric comorbidities than patients with PTD without TRD. Furthermore, median duration of TRD was approximately 30 times longer than that of PTD without TRD. Taken together, these results suggest that the cost of medical resources for the treatment of depression and comorbidities during PTD would be much higher in patients with TRD than PTD without TRD, as a previous study has reported [8].

As a first-line regimen in PTD, TCA and TeCA together were the most frequently used antidepressants followed by SSRI. This finding is not consistent with other studies that identified SSRIs as the most common first-line medication in both TRD and PTD without TRD [8, 18, 31]. The guidelines from the National Institute for Health and Clinical Excellence (NICE) for the treatment of depression in adults summarized that when compared with other antidepressants, TCA is equally effective, but less tolerable in outpatient clinics [34]. Although the reason for the preferred use of TCA and TeCA in South Korea cannot be explained from this study, the high drop-out rates in the first medication trial (i.e., 57% of the population had no regimen at all) might be partially caused by the intolerable features of TCA. Furthermore, the prescription frequency of serotonin antagonist and reuptake inhibitors (SARI), including trazodone, as a single regimen in the first- and second-line regimen, was higher than that of selective norepinephrine reuptake inhibitors (SNRI), norepinephrine-dopamine reuptake inhibitors (NDRI), and noradrenergic and specific serotonergic antidepressants (NaSSA). This can be likely explained by the sedative effect of SARI. In TRD, we hypothesized that if a regimen of SSRIs failed, the choice of follow-up medication would not include SSRIs, as observed in previous studies [8, 18]. However, the proportion of SSRI usage fluctuated with the order of regimens, i.e., when a regimen failed, there was no specific pattern for choosing the next antidepressant.

The definition of a regimen required a minimum number of days of medication supply, however, there has been no absolute standard for this. While we decided to set the threshold to 28 days based on clinical experiences, it is a limitation of this study nonetheless. To address this issue, we repeated our analyses by changing the minimum days of medication supply to 14, 28, and 42 days. To our knowledge, this is the first study which showed the differences of the proportions of TRD to PTD following three different minimum requirements of medication supply days. The proportion of TRD to PTD in the 28-days-set was approximately 0.63 times lower than that in the 14-day-set (4.17% / 6.61%), and 1.56 times higher than that in the 42-day-set (4.17% / 2.68%).

In contrast to previous studies [8, 18, 31], the current study attempted to consider not only the regimen of antidepressants, but also the regimen of antipsychotics. However, the minimum dosage requirement was applied only to the regimen of antipsychotics. This manipulation was an effort to reflect clinical practices, however, interpreting the results of this analysis may be more difficult.

In defining TRD, if no limitation is placed on the time of PTD onset to TRD onset, a higher prevalence is reported. Fife and colleagues reported a TRD rate of 20.94% among PTD cases and the time from onset of PTD to onset of TRD was longer than a year [18]. The authors assumed that this high rate of TRD proportion and prolonged period of treatment resulted from including not only acute treatment but also prophylactic treatment. Therefore, the authors excluded TRD cases whose time from onset of PTD to onset of TRD was longer than 6 months, which resulted in the proportion of TRD to PTD falling to 2.47%. We agreed that long-term maintenance treatment may reflect prophylaxis rather than acute treatment, therefore, we applied a rule that if a regimen lasted more than 90 days without failure, we considered it as an effective regimen and defined that the regimen is impossible to fail even if the regimen is followed by a new regimen. However, it is also a limitation of this study as the rule of 90 days is dichotomously strict and lacks research evidence.

To our knowledge, this is the first study which targeted approximately the entire population of South Korea to describe the epidemiology of PTD and TRD. However, it only provides a glimpse at the treatment of depression, due to limitations regarding the nature of claims data analysis. We built a rigorous and detailed model based on diagnoses and prescriptions to predict the characteristics of depressive disorders, nevertheless, constant gaps remain between predictions and patients’ real experiences.

## Supporting information

S1 Table The ICD-10 codes of psychiatric and non-psychiatric comorbidities. (PDF)

S2 Table Top 10 diagnoses of subjects who had a prescription of antidepressants but had not received a diagnosis of depression within 30 days from the day of disposal of antidepressants. (PDF)

S3 Table 14-day-set of prevalence of PTD and prevalence, proportion, incidence of TRD according to age and sex. (PDF)

S4 Table 42-day-set of prevalence of PTD and prevalence, proportion, incidence of TRD according to age and sex. (PDF)

S5 Table Psychiatric and non-psychiatric comorbidities in patients with PTD, PTD without TRD and TRD. (PDF)
