# Supplementary material for: Epidemiology of pharmaceutically treated depression and treatment resistant depression in South Korea
Source: PLoS One. 2019 Aug 23;14(8):e0221552. doi: 10.1371/journal.pone.0221552 (PMC6707549; doi:10.1371/journal.pone.0221552)
Supplement: S1 Table — (PDF) [file pone.0221552.s001.pdf]

Comorbidities	The ICD-10 codes
Psychiatric comorbidities	
Anxiety disorders	F40, F41, F43.1
Substance related disorders	F10~ F19
Obsessive-compulsive disorder	F42
Personality disorders	F60~ F69
Non-psychiatric comorbidities	
Cardiovascular diseases	I10~I15, I20~ I25, I47~~ I50,
Diabetes mellitus	E10~E14
Chronic obstructive pulmonary diseases	J40~J47
Cancer	C00~C97, D00~D09, D10~D36, D37~D48
Stroke	I60~I69
Hypothyroidism	E00~ E03

S1 Table . The ICD-10 codes of psychiatric and non-psychiatric comorbidities.
