# Supplementary material for: Epidemiology of pharmaceutically treated depression and treatment resistant depression in South Korea
Source: PLoS One. 2019 Aug 23;14(8):e0221552. doi: 10.1371/journal.pone.0221552 (PMC6707549; doi:10.1371/journal.pone.0221552)
Supplement: S2 Table — (PDF) [file pone.0221552.s002.pdf]

Diagnosis	Number of subjects (%)
	n = 416,836
F41 (Other anxiety disorders)	46,330 (11.1)
K21 (Gastro-oesophageal reflux disease)	16,696 (4.0)
I10 (Essential(primary) hypertension)	13,915 (3.3)
M54 (Dorsalgia)	12,527 (3.0)
K29 (Gastritis and duodenitis)	11,516 (2.8)
G44 (Other headache syndromes)	10,392 (2.5)
E11 (Type 2 diabetes mellitus)	10,349 (2.5)
F51 (Nonorganic sleep disorders)	10,238 (2.5)
F45 (Somatoform disorders)	9,382 (2.3)
I63 (Cerebral infarction)	9,046 (2.2)

S2 Table. Top 10 diagnoses of subjects who had a prescription of antidepressants but had not received a diagnosis of depression within 30 days from the day of disposal of antidepressants.
