# Supplementary material for: Epidemiology of pharmaceutically treated depression and treatment resistant depression in South Korea
Source: PLoS One. 2019 Aug 23;14(8):e0221552. doi: 10.1371/journal.pone.0221552 (PMC6707549; doi:10.1371/journal.pone.0221552)
Supplement: S3 Table — (PDF) [file pone.0221552.s003.pdf]

Group	General population	PTD cases	PTD Prevalence (%)	TRD cases	TRD Prevalence (%)	Proportion of TRD to PTD (%)	TRD Incidence (no. of event/100PY, 95% CI)
All subject	41,256,396	834,694	2.02	55,151	0.13	6.61	19.3 (19.1,19.5)
Male	20,464,613	290,206	1.42	18,635	0.09	6.42	17.9 (17.6,18.1)
18 – 29	4,219,688	28,605	0.68	2,277	0.05	7.96	31.9 (30.6,33.2)
30 – 39	4,163,684	36,320	0.87	2,272	0.06	6.26	24.3 (23.3,25.3)
40 – 49	4,510,316	51,579	1.14	3,548	0.08	6.88	21.6 (20.9,22.3)
50 – 59	3,915,566	66,878	1.71	4,390	0.11	6.56	17.7 (17.1,18.2)
60 – 69	2,083,585	54,487	2.62	3,378	0.16	6.20	14.0 (13.6,14.5)
70 – 79	1,246,286	41,641	3.34	2,346	0.19	5.63	12.8 (12.3,13.4)
≥ 80	325,488	10,696	3.29	424	0.13	3.96	10.5 (9.6,11.6)
Female	20,791,783	544,488	2.62	36,516	0.18	6.71	20.1 (19.9,20.3)
18 – 29	3,831,374	45,223	1.18	3,213	0.08	7.10	35.3 (34.1,36.5)
30 – 39	4,005,259	62,259	1.55	5,368	0.13	8.62	32.0 (31.1,32.8)
40 – 49	4,325,842	92,647	2.14	6,585	0.15	7.11	24.4 (23.8,25.0)
50 – 59	3,876,004	134,871	3.48	9,069	0.23	6.72	21.2 (20.8,21.7)
60 – 69	2,244,951	100,667	4.48	6,580	0.29	6.54	16.4 (16.0,16.8)
70 – 79	1,724,057	83,637	4.85	4,830	0.28	5.77	13.4 (13.0,13.7)
≥ 80	784,296	25,184	3.21	871	0.11	3.46	8.8 (8.3,9.4)
Male : Female	1 : 1.016	1 : 1.88	1 : 1.85	1:1.96	1:1.31	1:1.05	1:1.12

S3 Table. 14-day-set of prevalence of PTD and prevalence, proportion, incidence of TRD according to age and sex.
