# Supplementary material for: Epidemiology of pharmaceutically treated depression and treatment resistant depression in South Korea
Source: PLoS One. 2019 Aug 23;14(8):e0221552. doi: 10.1371/journal.pone.0221552 (PMC6707549; doi:10.1371/journal.pone.0221552)
Supplement: S4 Table — (PDF) [file pone.0221552.s004.pdf]

Group	General population	PTD cases	PTD Prevalence (%)	TRD cases	TRD Prevalence (%)	Proportion of TRD to PTD (%)	TRD Incidence (no. of event/100PY, 95% CI)
All subject	41,256,396	834,694	2.02	22,400	0.05	2.68	7.1 (7.0,7.2)
Male	20,464,613	290,206	1.42	7,731	0.04	2.66	6.8 (6.7,7.0)
18 – 29	4,219,688	28,605	0.68	965	0.02	3.37	12.0 (11.3,12.8)
30 – 39	4,163,684	36,320	0.87	969	0.02	2.67	9.3 (8.8,9.9)
40 – 49	4,510,316	51,579	1.14	1,529	0.03	2.96	8.4 (8.0,8.8)
50 – 59	3,915,566	66,878	1.71	1,924	0.05	2.88	7.1 (6.8,7.4)
60 – 69	2,083,585	54,487	2.62	1,366	0.07	2.51	5.3 (5.0,5.5)
70 – 79	1,246,286	41,641	3.34	838	0.07	2.01	4.3 (4.0,4.6)
≥ 80	325,488	10,696	3.29	140	0.04	1.31	3.3 (2.8,3.9)
Female	20,791,783	544,488	2.62	14,669	0.07	2.69	7.3 (7.2,7.4)
18 – 29	3,831,374	45,223	1.18	1,279	0.03	2.83	12.4 (11.7,13.1)
30 – 39	4,005,259	62,259	1.55	2,274	0.06	3.65	11.8 (11.3,12.3)
40 – 49	4,325,842	92,647	2.14	2,808	0.07	3.03	9.3 (8.9,9.6)
50 – 59	3,876,004	134,871	3.48	3,752	0.10	2.78	7.9 (7.6,8.1)
60 – 69	2,244,951	100,667	4.48	2,544	0.11	2.53	5.8 (5.5,6.0)
70 – 79	1,724,057	83,637	4.85	1,735	0.10	2.07	4.4 (4.2,4.6)
≥ 80	784,296	25,184	3.21	277	0.04	1.10	2.7 (2.4,3.0)
Male : Female	1 : 1.016	1 : 1.88	1 : 1.85	1:1.90	1:1.31	1:1.01	1:1.07

S4 Table. 42-day-set of prevalence of PTD and prevalence, proportion, incidence of TRD according to age and sex.
