# Supplementary material for: Epidemiology of pharmaceutically treated depression and treatment resistant depression in South Korea
Source: PLoS One. 2019 Aug 23;14(8):e0221552. doi: 10.1371/journal.pone.0221552 (PMC6707549; doi:10.1371/journal.pone.0221552)
Supplement: S5 Table — (PDF) [file pone.0221552.s005.pdf]

	PTD (%)	PTD without TRD (%)	TRD (%)	*p*-value [a]
All subject	834,694 (100.0)	799,882 (100.0)	34,812 (100.0)	
Psychiatric comorbidities				
Anxiety disorders	245,401 (29.4)	220,624 (27.6)	24,777 (71.1)	<.0001
Substance use disorders	27,354 (3.3)	23,345 (2.9)	4,009 (11.5)	<.0001
Obsessive-compulsive disorder	6,951 (0.8)	5,862 (0.7)	1,089 (3.1)	<.0001
Personality disorders	4,928 (0.6)	4,140 (0.5)	788 (2.3)	<.0001
Non-psychiatric comorbidities				
Cardiovascular diseases	244,228 (29.3)	227,162 (28.4)	17,066 (49.0)	<.0001
Diabetes mellitus	138,701 (16.6)	126,926 (15.9)	11,775 (33.8)	<.0001
Chronic obstructive pulmonary diseases	126,481 (15.2)	111,349 (13.9)	15,132 (43.4)	<.0001
Cancer	81,961 (9.8)	72,609 (9.1)	9,352 (26.9)	<.0001
Stroke	81,173 (9.7)	74,754 (9.3)	6,419 (18.4)	<.0001
Hypothyroidism	31,327 (3.8)	26,922 (3.4)	4,405 (12.7)	<.0001

S5 Table. Psychiatric and non-psychiatric comorbidities in patients with PTD, PTD without TRD and TRD.

[a] Chi-squared tests were performed between PTD without TRD and TRD
